# Neuregulin-1β increases glucose uptake and promotes GLUT4 translocation in palmitate-treated C2C12 myotubes by activating PI3K/AKT signaling pathway

**DOI:** 10.3389/fphar.2022.1066279

**Published:** 2023-01-10

**Authors:** Meirong Yu, Shuang Wu, Chao Gong, Lianhua Chen

**Affiliations:** Department of Anesthesiology, Shanghai General Hospital, Shanghai Jiao Tong University School of Medicine, Shanghai, China

**Keywords:** Neuregulin-1β, C2C12 myotubes, glucose uptake, GLUT4 translocation, PI3K/AKT

## Abstract

Insulin resistance (IR) is a feature of type 2 diabetes (T2DM) accompanied by reduced glucose uptake and glucose transporter 4 (GLUT4) translocation by skeletal muscle. Neuregulin-1β (NRG-1β) is essential for myogenesis and the regulation of skeletal muscle metabolism. Neuregulin-1β increases insulin sensitivity, promotes glucose uptake and glucose translocation in normal skeletal muscle. Here, we explored whether Neuregulin-1β increased glucose uptake and GLUT4 translocation in palmitate (PA)-treated C2C12 myotubes. After C2C12 myoblasts differentiated into myotubes, we used palmitate to induce cellular insulin resistance. Cells were incubated with or without Neuregulin-1β and glucose uptake was determined using the 2-NBDG assay. The expression level of glucose transporter 4 (GLUT4) was measured *via* immunofluorescence and Western blotting. MK2206, an inhibitor of AKT, was employed to reveal the important role played by AKT signaling in PA-treated C2C12 myotubes. We then established an animal model with T2DM and evaluated the effects of Neuregulin-1β on body weight and the blood glucose level. The GLUT4 level in the gastrocnemius of T2DM mice was also measured. NRG-1β not only increased glucose uptake by PA-treated myotubes but also promoted GLUT4 translocation to the plasma membrane. The effect of NRG-1β on PA-treated C2C12 myotubes was associated with AKT activation. In T2DM mice, Neuregulin-1β not only improved diabetes-induced weight loss and diabetes-induced hyperglycemia, but also promoted GLUT4 translocation in the gastrocnemius. In summary, Neuregulin-1β increased glucose uptake and promoted translocation of GLUT4 to the plasma membrane in PA-treated C2C12 myotubes by activating the PI3K/AKT signaling pathway.

## 1 Introduction

The incidence of type 2 diabetes (T2DM) is increasing in both developing and developed countries. The International Diabetes Federation (IDF) Diabetes Atlas indicates that the global diabetes prevalence in those aged 20–79 years in 2021 was 10.5% (536.6 million people), and is expected to rise to 12.2% (783.2 million) in 2045 (Sun et al., 2022). T2DM is closely associated with insulin resistance (IR), a pathophysiological condition characterized by impaired insulin action on insulin-sensitive tissues, including skeletal muscle, liver and adipose tissue (Al-Sulaiti et al., 2019; Hagman et al., 2019). The principal features of IR in insulin-sensitive tissues (including skeletal muscle) are decreases in glucose uptake and glucose transporter 4 (GLUT4) translocation to the plasma membrane (Luo et al., 2019; Sylow et al., 2021). Skeletal muscle accounts for 40% of body weight, and is responsible for up to 70%–90% of insulin-mediated glucose disposal under normal physiological conditions (Liu et al., 2020; Merz and Thurmond, 2020; Feraco et al., 2021). Indeed, skeletal muscle IR is considered as the most important extra-pancreatic factor in T2DM pathogenesis (Song et al., 2013; Yaribeygi et al., 2019; Galicia-Garcia et al., 2020). Thus, improving the insulin sensitivity of skeletal muscle is important to control T2DM progression.

Neuregulin-1 (NRG-1) is a member of a large family of epidermal growth factor (EGF) proteins. Many different isoforms are known; all share an EGF-like domain that mediates biological activity (Falls, 2003). Different NRG-1 isoforms may have distinct tissue specificity and receptor affinity, and NRG-1β was used to study the effects of NRG-1 on skeletal muscle cell in previous studies (Suárez et al., 2001; Canto et al., 2004; Cantó et al., 2006). NRG-1β plays an important role in cell survival, proliferation, migration and differentiation (Falls, 2003; Guma et al., 2010). NRG-1β increased insulin sensitivity, promoted glucose uptake and glucose transporter translocation in normal skeletal muscle (independent of the insulin level) (Suárez et al., 2001; Canto et al., 2004; Cantó et al., 2007; Ennequin et al., 2017). However, there are few reports on whether insulin sensitivity can still be improved in the IR environment by NRG-1β. NRG-1β increases glucose uptake by activating the PI3K/AKT pathway in cardiomyocyte (Pentassuglia et al., 2016; Heim et al., 2020), which can promote GLUT4 translocation (Zhu et al., 2013; Sharma et al., 2015; Li et al., 2016). Hence, NRG-1β may activate the PI3K/AKT pathway in palmitate (PA)-treated C2C12 myotubes.

In our study, we explored whether NRG-1β treatment protected against IR in PA-treated C2C12 myotubes and the molecular mechanism in play. We hypothesized that NRG-1β would increase glucose uptake and promote GLUT4 translocation by activating the PI3K/AKT pathway in PA-treated C2C12 myotubes. To verify this, we investigated the effect of NRG-1β on glucose uptake and GLUT4 translocation in PA-treated C2C12 myotubes, and the relationship between NRG-1β action and PI3K/AKT signaling. We found that NRG-1β treatment increased glucose uptake and GLUT4 translocation *via* the PI3K/AKT signaling pathway in PA-treated C2C12 myotubes.

## 2 Materials and methods

### 2.1 Cell culture, differentiation, and treatment

Mouse skeletal muscle cell lines (C2C12 myoblasts) were obtained from Shanghai FuHeng Biology (Shanghai, China). The cells were cultured in Dulbecco’s modified Eagle’s medium (DMEM; Gibco Co., Ltd., Grand Island, NY, United States) containing 10% (v/v) fetal bovine serum (Gibco) and 1% (w/v) penicillin–streptomycin (P/S; NCM Biotech, Suzhou, China) in a humidified incubator under 5% (v/v) CO_2_ at 37°C. To initiate differentiation, cells were grown to 70%–80% confluence, and then incubated with DMEM with 2% (v/v) heat-inactivated horse serum (Gibco) and 1% (w/v) P/S for 4–6 days. The medium was changed daily. The fully differentiated myotubes were used in the following experiments.

### 2.2 Cell viability assessment

C2C12 myoblasts were seeded in 96-well plates and grown for differentiation. After the formation of myotubes, 0, .25, .5, .75 or 1 mM PA was added for 24 h, and then cell viability was measured using cell counting kit-8 (Beyotime) according to the manufacturer’s protocol. Next, the absorbance was measured at a wavelength of 450 nm using Microplate Reader (Thermo Fisher Scientific). Cell viability was expressed as a percentage of the optical density of each treatment group relative to the control group.

### 2.3 Palmitate-induced insulin resistance in C2C12 myotubes

Firstly, palmitate (33.40mg, Sigma, St. Louis, MO, United States) was dissolved in 3 mL double distilled water (v/v) with heating at 75°C. Next, preparation 40% free fatty acid-free bovine serum albumin solution (BSA), fatty acid free BSA powder (1.2 g, Beyotime, Shanghai, China) dissolved in phosphate-buffered saline (PBS), centrifugated at room temperature 8,000 rpm for 15 min until BSA completely dissolved, and then fixed to 3 mL with PBS. Thirdly, mixing the two liquids together to make a 20 mM solution and then sterilized by passing through a .22-μm-pore-sized filter, and this solution was stored at −20°C and used within 2 weeks. Fully differentiated myotubes were treated with .25 mM PA for 24 h (Petersen and Shulman, 2018; Shen et al., 2019). Control cells were treated with the same volume of PBS–BSA DMEM with or without insulin. PA-treated C2C12 myotubes were serum-depleted for 4.5 h and then incubated with or without 10 ng/mL NRG-1β (PeproTech, Rocky Hill, NJ, United States) for 1.5 h (Suárez et al., 2001; Canto et al., 2004; Heim et al., 2020). After 1 h treatment of NRG-1β, cells were then incubated with or without insulin (100 nM) for 30 min.

### 2.4 Glucose uptake assay

Glucose uptake by differentiated C2C12 myotubes was measured using the 2-NBDG assay that employs a fluorescent D-glucose analogue (Zou et al., 2005; Bala et al., 2021). After incubation with or without NRG-1β, cells were incubated with 80 µM 2-NBDG (APExBIO, Houston, TX, United States) at 37°C for 30 min 2-NBDG uptake was stopped by removing the incubation medium and washing the cells with 1×PBS followed to remove free 2-NBDG. And then discarding the PBS, the cells were subsequently resuspended in pre-cool RIPA (Beyotime) and transferred to 96-well culture plates (Xu et al., 2018). Next, 2-NBDG levels were determined using a fluorescence microplate (excitation 485 nm, emission 535 nm; Thermo Fisher Scientific, Massachusetts, MA, United States). And we were very careful to avoid light during the experiment.

### 2.5 Animal model

The animal protocol was approved (Protocol number: 2019-A005-1, Shanghai, China) by the Shanghai General Hospital Clinical Center Laboratory Animal Welfare and Ethics Committee. Thirty pathogen-free 3–4-week-old male C57BL/6J wild-type mice (SLAC, Shanghai, China) (18–20 g) were used. The animals were housed in a temperature-controlled (25°C ± 2°C) room under a 12 h light/dark cycle with a relative humidity of 50%–70%. T2DM was induced as described previously (Hamza et al., 2011; Kleinert et al., 2018; Yang et al., 2020). The mice were randomly divided into a control group (CON, *n* = 10), a T2DM + PBS group (DM, n = 10) and a T2DM + NRG-1β group (DM + NRG-1β, *n* = 10). All mice were acclimatized for 1 week prior to experimentation. The CON group was fed normal chow; the DM and the DM + NRG-1β groups were fed a high-fat diet [high fat (60FDC) Purified Rodent Diet; D12492; Research Diets, New Brunswick, NJ, United States) for five consecutive weeks. Then, after 12 h of fasting, the DM and DM + NRG-1β groups were intraperitoneally injected with 50 mg/kg streptozocin (STZ) (S0130; Sigma, St. Louis, MO, United States) daily for 3 days. The CON group was intraperitoneally injected with isometric citric acid buffer. The blood glucose levels were measured 7 days after the final injection. When that level was >16.7 mmol/L, T2DM was considered established (Kleinert et al., 2018; Yang et al., 2020). DM + NRG-1β group and DM group received 30 μg/kg NRG-1β (*via* intraperitoneal injection) or the same volume of PBS weekly for 4 weeks, respectively (Ennequin et al., 2015; Wu et al., 2019; Liu et al., 2020). After a week final treatment of NRG-1β, mice were anesthetized with 1% (w/v) pentobarbital sodium and euthanized by decapitation. The gastrocnemius tissues were dissected, weighed, and soaked overnight at 4°C in 4% (v/v) paraformaldehyde (PFA) and then in 30% (w/v) sucrose at 4°C for 48 h to immunofluorescence analysis or frozen in liquid nitrogen and stored at −80°C for later biochemical analysis.

### 2.6 Western blotting

After washing with ice-cold PBS, cells or gastrocnemius tissues were homogenized. Total protein was extracted into pre-cooled RIPA lysis buffer (Beyotime) with 1% (w/v) of a protease/phosphatase inhibitor cocktail (NCM Biotech) at 4°C for 15 min, and the protein concentration was measured using a BCA protein assay kit (Beyotime). Protein loading buffer (5X) (NCM Biotech) was added to the lysates followed by protein denaturation by placing the tubes in boiling water for 10 min.

Membrane proteins (including GLUT4) were obtained using Membrane and Cytosol Protein Extraction Kit (Beyotime), according to the manufacturer’s instructions. Western blotting was employed to determine GLUT4 expression levels on plasma membranes. The membrane marker Na^+^-K^+^-ATPase served as a control.

Proteins (20–30 μg/lane) were subjected to 10% (v/v) sodium dodecyl sulphate polyacrylamide gel electrophoresis (Epizyme, Suzhou, China) and transferred to polyvinylidene fluoride membranes (Immobilon P; Millipore, Billerica, MA, United States) and the blots saturated with blocking buffer (NCM Biotech) for 15 min at room temperature and then incubated overnight at 4°C with antibodies against β-actin (1:5,000; Proteintech, Wuhan, China), myosin heavy chain (MHC; 1:1,000; Proteintech), Phosphor-Akt (ser 473) (1:1,000; Cell Signaling Technology, Massachusetts, MA, United States), AKT (1:1,000; Proteintech), GLUT4 (1:1,000; Cell Signaling Technology) and Na^+^-K^+^-ATPase (1:1,000; Cell Signaling Technology). The gray intensity of protein was measured using ImageJ software (United States National Institutes of Health). Three independent experiments were performed and the data averaged.

### 2.7 Immunofluorescence

C2C12 myoblasts were cultured on 35-mm-diameter confocal dishes and allowed to differentiate for 4–5 days. After treatment with NRG-1β, the myotubes were fixed in 4% (v/v) PFA for 15 min, permeabilized by adding a buffer containing saponin (Beyotime) for 10 min at room temperature, and then incubated in PBS with 3% (w/v) BSA for 1 h at room temperature, followed by incubation with anti-GLUT4 (1:300; Affinity, Suzhou, China) overnight at 4°C. The cells were washed three times with PBS (10 min each time) and then incubated with Alexa Fluor 594-conjugated goat anti-mouse secondary antibody for 2 h at room temperature. Detection was performed using the confocal microscope (TCS SP8 X; Leica Heidelberg, Germany).

Mice were anesthetized with 1% (w/v) pentobarbital sodium and perfused with saline for a few minutes, followed by perfusion with 4% (v/v) PFA in PBS. The gastrocnemius tissues were dissected and soaked overnight at 4°C in 4% (v/v) PFA and then in 30% (w/v) sucrose at 4°C for 48 h. Gastrocnemius slices (20 μm) were prepared and immunofluorescence detected as described above.

### 2.8 Statistical analysis

All experiments were repeated at least three times. Data are presented as means ± standard error of the mean (SEM) and were compared *via* one-way analysis of variance. *p*-values <.05 were considered statistically significant.

## 3 Results

### 3.1 NRG-1β increased glucose uptake in PA-treated C2C12 myotubes

**FIGURE 1:**
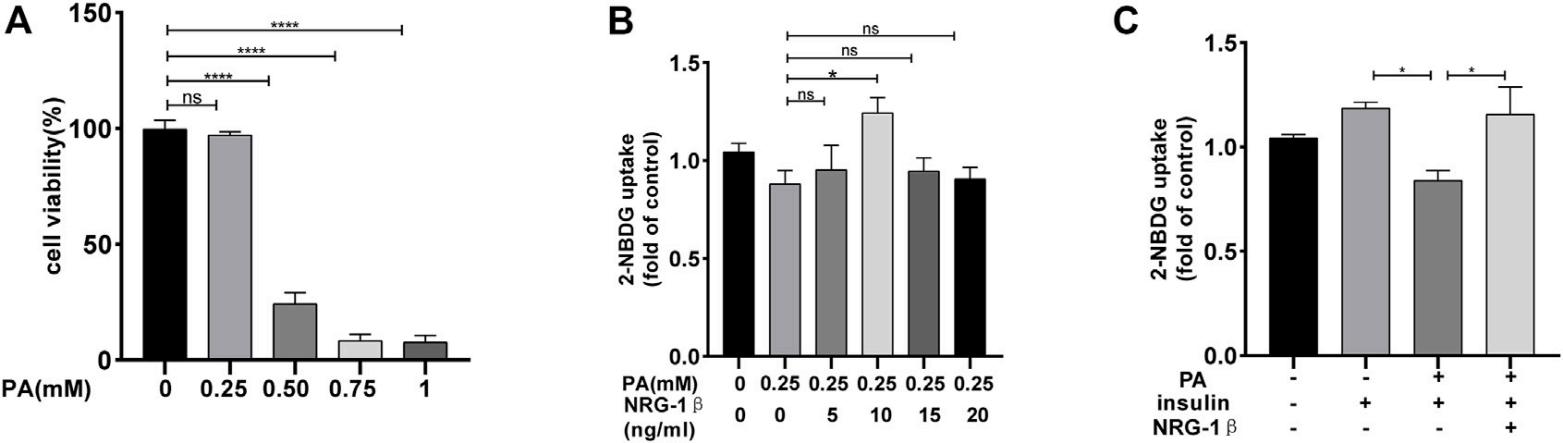
NRG-1β increased glucose uptake in PA-treated C2C12 myotubes. C2C12 myotubes were incubated with different concentrations of PA for 24 h and cell viability measured using the CCK8 assay **(A)**. C2C12 myotubes were serum depleted for 4.5 h and then incubated for 1.5 h with different concentrations of NRG-1β (0, 5, 10, 15, and 20 ng/mL) **(B)**. C2C12 myotubes were incubated in the presence or absence of the indicated concentration (10 ng/mL) of NRG-1β for 1.5 h, the cells were incubated with 80 μM 2-NBDG for 30 min. The 2-NBDG uptake was determined as described in the Methods **(C)**. The data are presented as the mean ± SEM. ns, *p* > .05; ^*^ *p* < .05; ^****^ *p* < .0001.

C2C12 myoblasts differentiated into myotubes, as confirmed by the development of the myotube-like multi-nuclear structure (Supplementary Figures S1A, B) and a structure protein marker MHC (Supplementary Figures S1C, D). To explore the effects of NRG-1β on glucose uptake and GLUT4 translocation in IR environment, we reproduced the classic cellular model in the C2C12 myotubes (Nieuwoudt et al., 2017; Petersen and Shulman, 2018). Glucose uptake was measured with the aid of the 2-NBDG assay (Zou et al., 2005; Bala et al., 2021). PA at .25 mM did not affect C2C12 myotube cell viability (Figure 1A). And then, C2C12 myotubes were incubated with various concentrations of NRG-1β for 1.5 h (Suárez et al., 2001; Canto et al., 2004). NRG-1β at 10 ng/mL optimally increased glucose uptake (Figure 1B). Glucose uptake was decreased in PA-treated (plus insulin) C2C12 myotubes compared to BSA (plus insulin) group, but NRG-1β rescued the uptake (Figure 1C). Thus, NRG-1β increased glucose uptake in PA-treated C2C12 myotubes.

### 3.2 NRG-1β promoted GLUT4 translocation in PA-treated C2C12 myotubes

**FIGURE 2:**
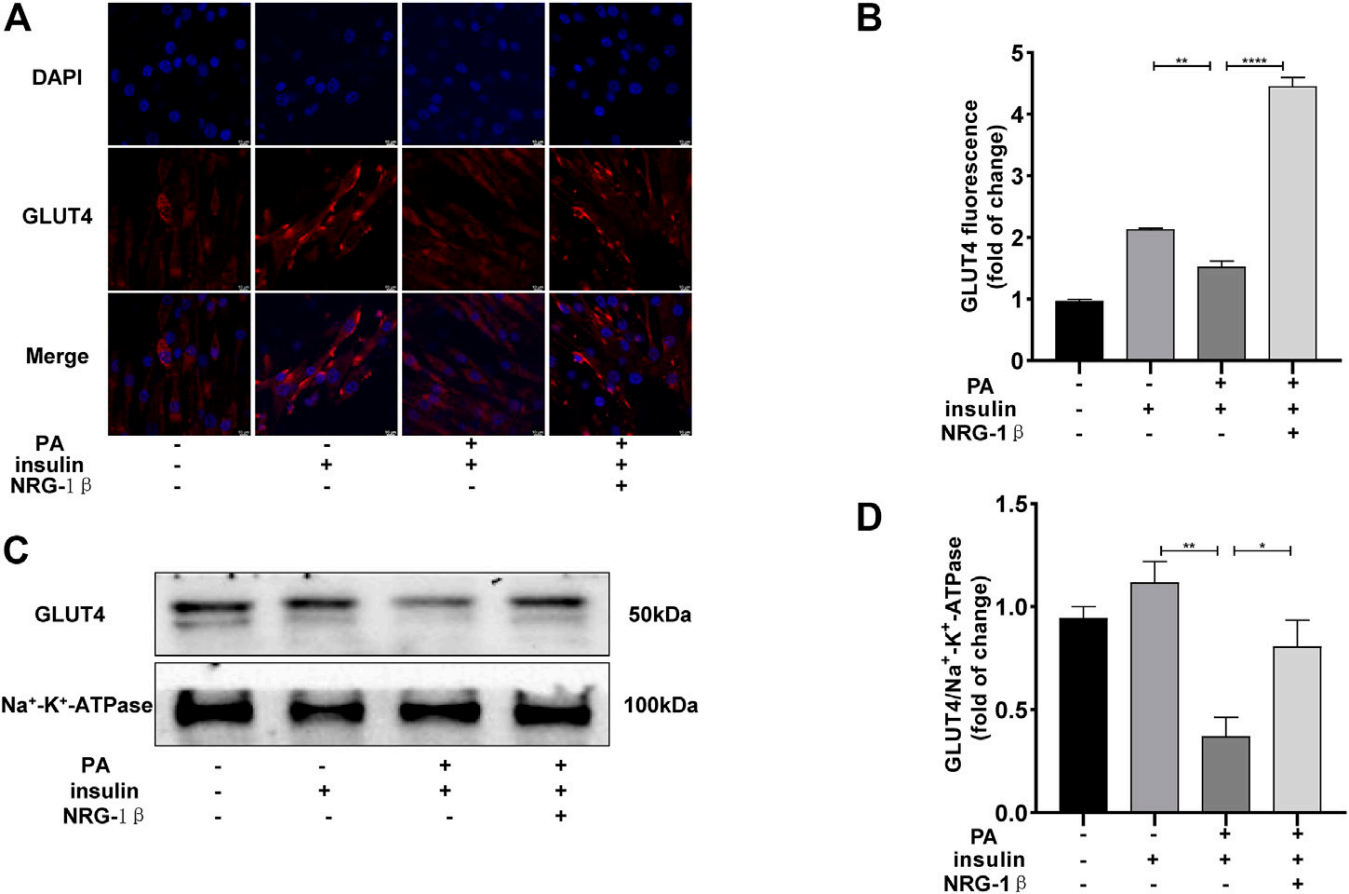
NRG-1β promoted the translocation of GLUT4 in PA-treated C2C12 myotubes. Effects of NRG-1β on GLUT4 translocation in PA-treated (plus insulin) C2C12 myotubes, GLUT4 was detected by using a fluorescent anti-GLUT4 antibody **(A,B)** (Scale bar: 10 μm) and Western blotting **(C,D)**. The data are presented as the mean ± SEM. ^*^ *p* < .05; ^**^ *p* < .01; ^****^ *p* < .0001.

To explore the effects of NRG-1β on GLUT4 translocation in PA-treated C2C12 myotubes, we used immunofluorescence staining to detect GLUT4. The GLUT4 levels were significantly decreased in PA-treated (plus insulin) C2C12 myotubes compared BSA (plus insulin) group; however, NRG-1β rescued the decrease (Figures 2A, B). Next, we examined cell membrane GLUT4 levels in the presence or absence of NRG-1β. GLUT4 membrane translocation decreased in PA-treated C2C12 myotubes, but NRG-1β rescued the fall (Figures 2C, D). Therefore, NRG-1β promoted GLUT4 translocation in PA-treated C2C12 myotubes.

### 3.3 AKT played an essential role in NRG-1β improving IR

**FIGURE 3:**
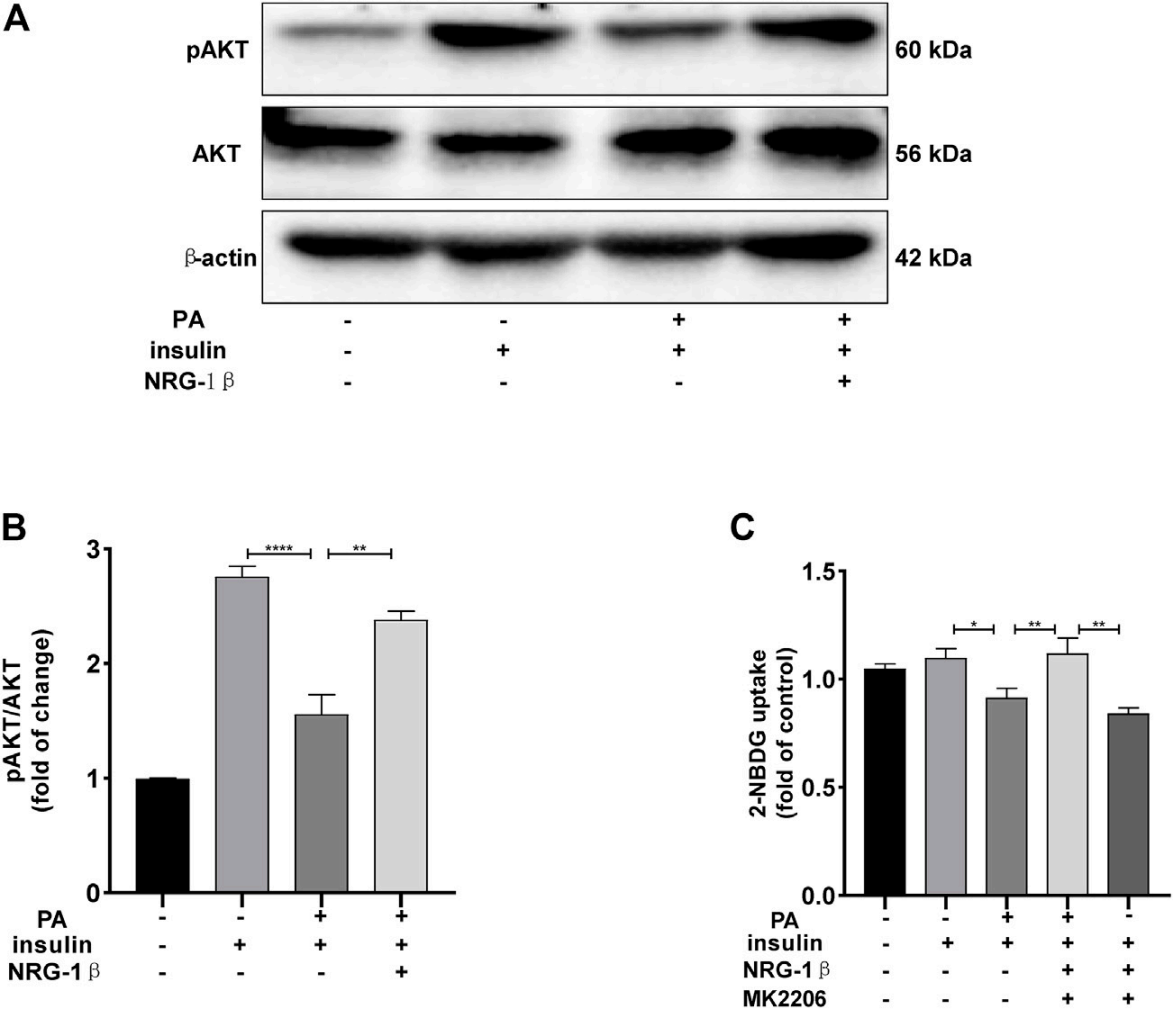
NRG-1β increased glucose uptake in PA-treated C2C12 myotubes by activating the PI3K/AKT signaling pathway. Effects of NRG-1β on levels of AKT and Phosphor-Akt (ser 473) in PA-treated (plus insulin) C2C12 myotubes were detected *via* Western blotting **(A,B)**. Dimethyl sulphoxide (DMSO) or 10 μM MK2206 (an AKT inhibitor) in DMSO was added before culturing .25 mM PA-treated (plus insulin) C2C12 myotubes, effects of MK2206 on glucose uptake using the 2-NBDG assay **(C)**. The data are presented as the mean ± SEM. ^*^ *p* < .05; ^**^ *p* < .01; ^****^ *p* < .0001.

**FIGURE 4:**
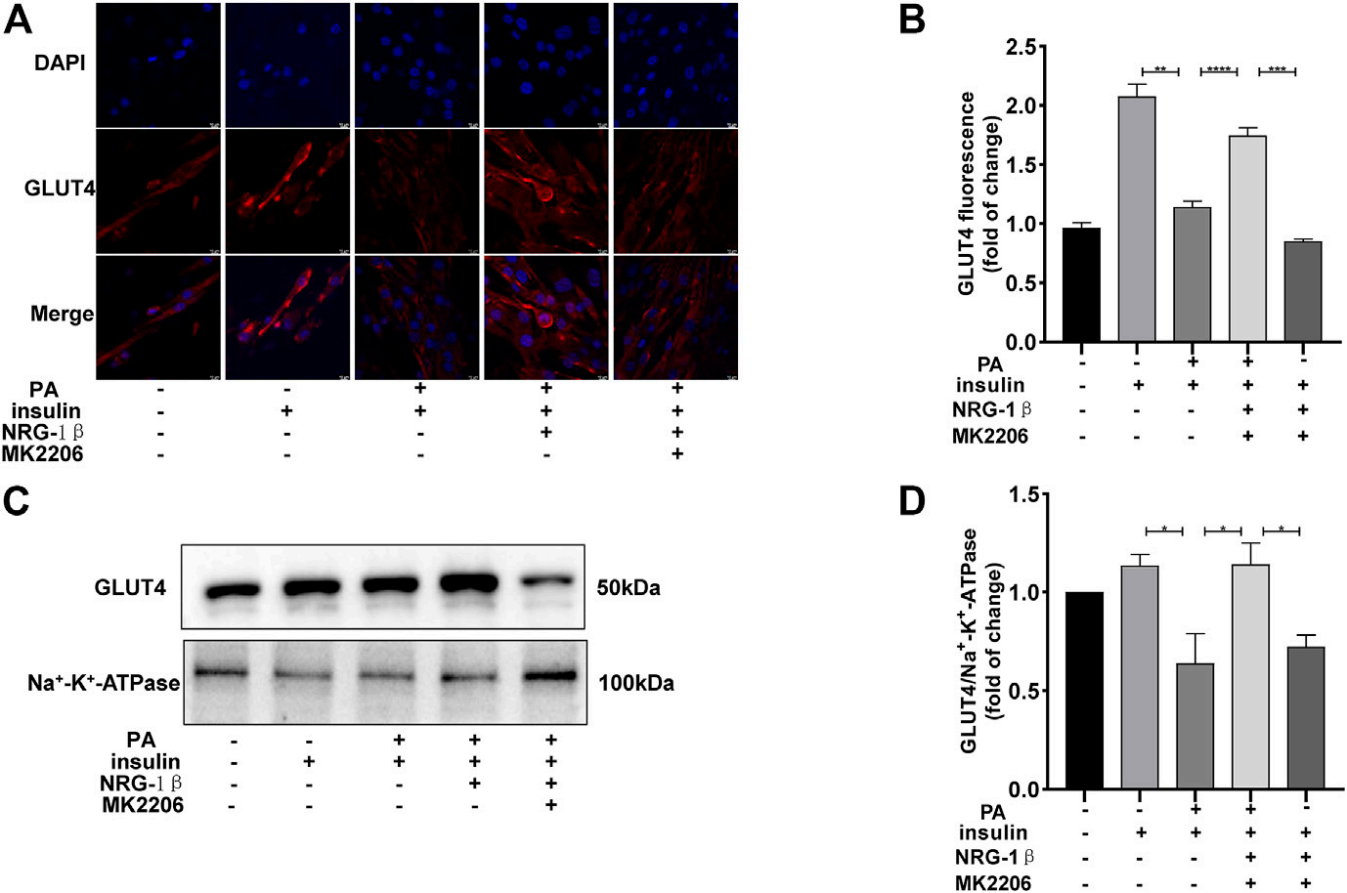
NRG-1β promoted the translocation of GLUT4 in PA-treated C2C12 myotubes by activating the PI3K/AKT signaling pathway. Effects of MK2206 of GLUT4 translocation in PA-treated C2C12 myotubes, GLUT4 was detected by using a fluorescent anti-GLUT4 antibody (Scale bar: 10 μm) **(A,B)** and Western blotting **(C,D)**. The data are presented as the mean ± SEM. ^*^ *p* < .05; ^**^ *p* < .01; ^***^ *p* < .001; ^****^ *p* < .0001.

The PI3K/AKT pathway triggers the GLUT4 translocation that is essential for glucose uptake and metabolism (Zhu et al., 2013; Li et al., 2017; Sharma and Dey, 2021). To explore the molecular mechanisms in play, we measured the AKT and phosphorylation of AKT protein levels. Phosphor-Akt (ser 473) expression was significantly decreased in PA-treated (plus insulin) C2C12 myotubes compared to the BSA (plus insulin) group, but NRG-1β increased the level significantly (Figures 3A, B), indicating that NRG-1β activated PI3K/AKT signaling in PA-treated C2C12 myotubes. Next, we determined the effects of pretreatment with MK2206 (an AKT inhibitor) on glucose uptake and translocation of GLUT4 to the plasma membrane of PA-treated C2C12 myotubes. Pretreatment with MK2206 significantly reduced the effects of NRG-1β on glucose uptake (Figure 3C) and GLUT4 translocation to the plasma membrane (Figure 4). These data suggested that NRG-1β increased glucose uptake and GLUT4 translocation in PA-treated C2C12 myotubes by activating PI3K/AKT signaling.

### 3.4 NRG-1β improved diabetes-induced weight loss and attenuated diabetes-induced hyperglycemia in T2DM mice

**FIGURE 5:**
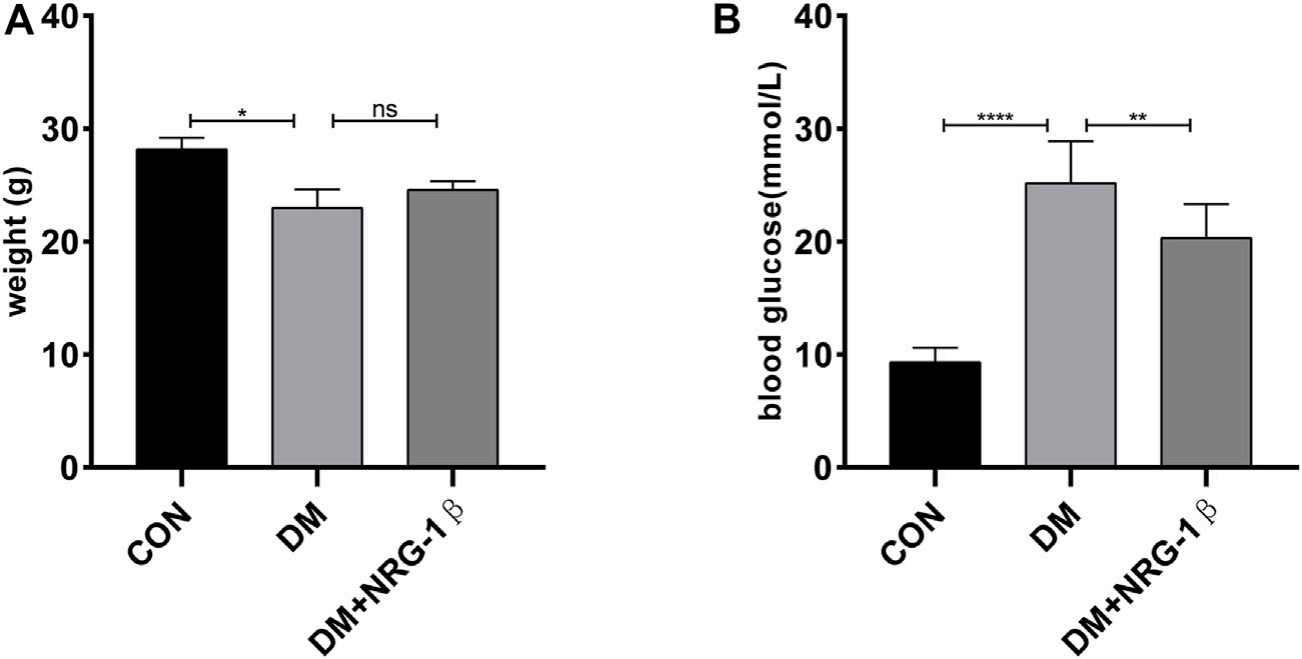
NRG-1β improved diabetes-induced weight loss and attenuated diabetes-induced hyperglycemia in T2DM mice. Effects of NRG-1β on the body weights **(A)** and blood glucose levels **(B)**. The data are presented as the mean ± SEM. ns, *p* > .05; *p < .05; **p < .01; ****p < .0001.

We established a T2DM animal model by combing high-fat diet with a low dose of STZ to evaluate the effects of NRG-1β. Seven days after the final intraperitoneal injection of STZ, establishment was successful (the blood glucose level was >16.7 mmol/L) (Hamza et al., 2011; Kleinert et al., 2018; Yang et al., 2020). The body weight of the DM group began to decrease after STZ injection, in contrast to the constant weight gain of the CON group, but NRG-1β improved diabetes-induced weight loss (Figure 5A). The blood glucose level remained high after STZ injection in the DM group; NRG-1β attenuated diabetes-induced hyperglycemia significantly (Figure 5B).

### 3.5 NRG-1β promoted the translocation of GLUT4 in the skeletal muscle of T2DM mice

**FIGURE 6:**
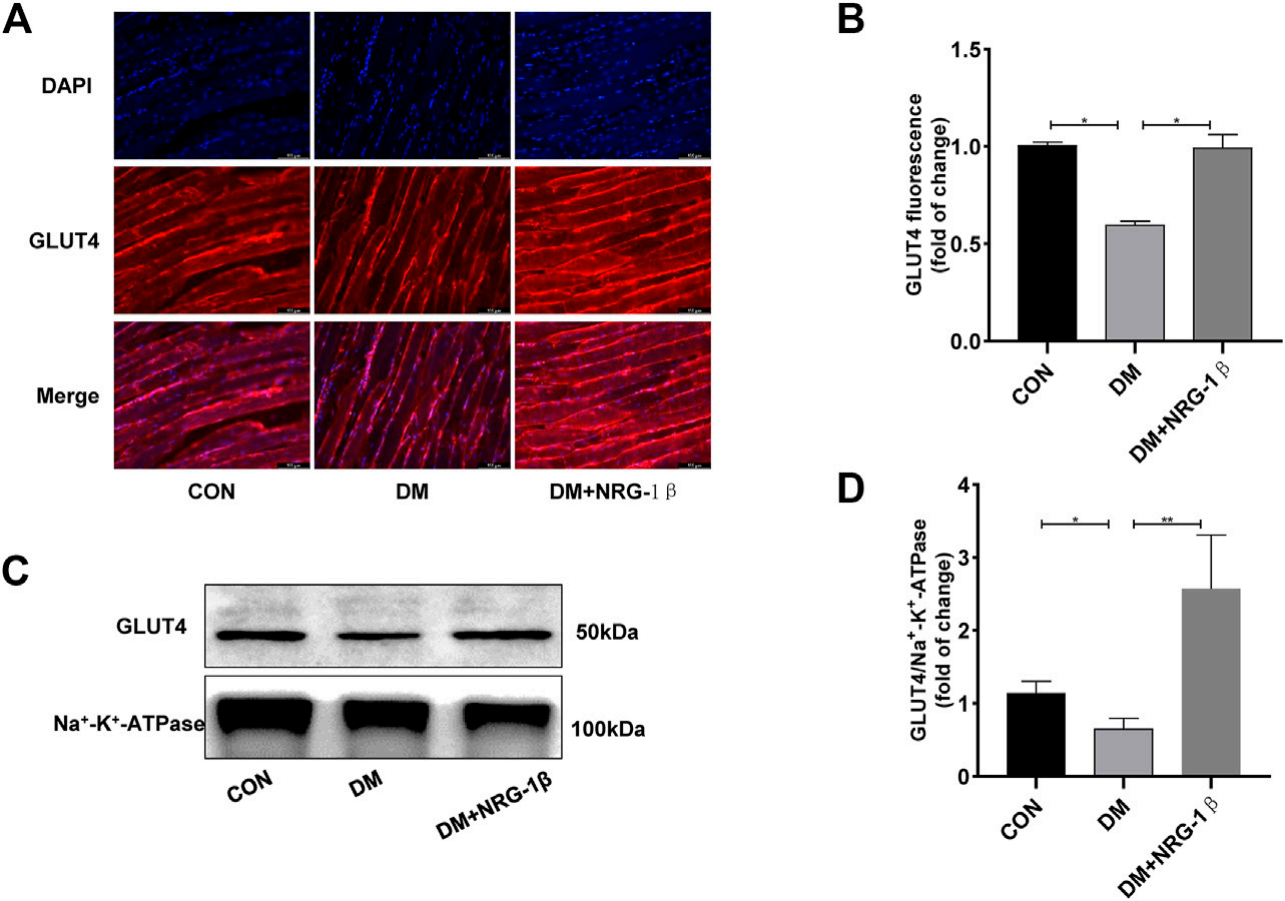
NRG-1β promoted the translocation of GLUT4 in the skeletal muscle of T2DM mice. Effects of NRG-1β on gastrocnemius GLUT4 translocation, GLUT4 was detected by using a fluorescent anti-GLUT4 antibody (Scale bar: 100 μm) **(A,B)** and Western blotting **(C,D)**. The data are presented as the mean ± SEM. ^*^ *p* < .05; ^**^ *p* < .01.

T2DM induced a remarkable change in the distribution of GLUT4 in skeletal muscle tissue (Fang et al., 2017; Fujiwara et al., 2017). After treatment with 30 μg/kg NRG-1β once weekly for 4 weeks, we examined the translocation of GLUT4 in gastrocnemius tissues in C57BL/6J and T2DM mice. The GLUT4 fluorescence intensity of T2DM mice were significantly lower than those of C57BL/6J mice of the same age; however, NRG-1β inhibited the reduction (Figures 6A, B). These observations were confirmed by Western blotting (Figures 6C, D).

## 4 Discussion

The principal function of insulin in the skeletal muscle is to promote cellular glucose uptake, a process controlled by GLUT4 translocation (Petersen and Shulman, 2018; Luo et al., 2019; Sylow et al., 2021). When skeletal muscle occurred IR, the action of insulin was impaired. In addition, skeletal muscle IR plays a central role in the pathogenesis of T2DM (Song et al., 2013). NRG-1β plays a key role in the development of skeletal muscle; it not only increased mitochondrial oxidative capacity and insulin sensitivity in normal skeletal muscle cells, but also improved complex 2-mediated mitochondrial respiration in the gastrocnemius of both control and diabetic mice (Suárez et al., 2001; Canto et al., 2004; Cantó et al., 2007; Ennequin et al., 2017). We initially studied the effects of NRG-1β on glucose uptake and GLUT4 translocation in PA-treated skeletal muscle cells, and then in T2DM mice. NRG-1β increased glucose uptake and promoted the translocation of GLUT4 in PA-treated C2C12 myotubes *via* the PI3K/AKT signaling pathway. Moreover, NRG-1β not only improved diabetes-induced weight loss and diabetes-induced hyperglycemia, but also increased the translocation of GLUT4 in the gastrocnemius in T2DM.

It has become increasingly apparent that elevated levels of plasma free fatty acids play an essential role in the impairment of insulin sensitivity characteristic of T2DM (Merz and Thurmond, 2020). Specifically, saturated fatty acids change the insulin biology and high consumption of PA (a saturated fatty acid) is associated with IR development. PA potently induces IR in cultured myocytes by impairing glucose uptake and reducing GLUT4 translocation in skeletal muscle, and PA treatment is commonly used to establish IR phenotype *in vitro* (Miller et al., 2009; Petersen and Shulman, 2018; Feraco et al., 2021; Sánchez-Alegría et al., 2021). We found that C2C12 myotubes treated with .25 mM PA remained fully viable, but evidenced decreases in glucose uptake and translocation of GLUT4 to the plasma membrane, as reported previously (Liu et al., 2020; Feraco et al., 2021). GLUT4 is a major mediator of glucose removal from the circulation to cell and a key regulator of whole-body glucose homeostasis (Huang and Czech, 2007). In the absence of stimulation, GLUT4 resides in cytoplasmic vesicles in a non-active state; only ∼1% of cellular GLUT4 is in the plasma membrane to implement transport function (Sadler et al., 2013; Yaribeygi et al., 2019). In response to stimulation, GLUT4 translocates to the cell membrane and facilitates glucose entry into cells, greatly lowering the blood glucose (Sadler et al., 2013; Yaribeygi et al., 2019). Reductions of GLUT4 translocation and activity affect glucose uptake, triggering IR; enhanced expression and translocation of GLUT4 improve IR in T2DM models (Watson and Pessin, 2001; Ariga et al., 2008; Yang et al., 2014). The GLUT4 level on the surface of muscle cells provoked systemic changes in glucose disposal *in vivo* (Huang and Czech, 2007). We thus explored the effect of NRG-1β on the cell surface GLUT4 level; NRG-1β rescued the reduction in GLUT4 translocation caused by PA. This is the first study to describe the effect of NRG-1β on glucose uptake and GLUT4 translocation in PA-treated C2C12 myotubes (Suárez et al., 2001; Canto et al., 2004; Pentassuglia et al., 2016; Heim et al., 2020).

Activation of the PI3K/AKT pathway (which is associated with GLUT4 translocation) is essential for glucose uptake and metabolism (Zhu et al., 2013; Sharma et al., 2015; Li et al., 2017; Miyata et al., 2017). AKT plays the predominant role in regulating glucose uptake by skeletal muscle; AKT knockdown decreases GLUT4 translocation, ultimately causing IR (Sharma and Dey, 2021; Sylow et al., 2021). In skeletal myotubes, PA affects the insulin-mediated activation of AKT (Schmitz-Peiffer et al., 1999). Thus, we explored whether NRG-1β improved IR in PA-treated C2C12 myotubes *via* the PI3K/AKT signaling pathway. We found that NRG-1β increased the expression of phosphor-Akt in PA-treated C2C12 myotubes. We used the AKT inhibitor MK2206 to explore the role played by AKT signaling. As predicted, pretreatment with MK2206 significantly reduced the effects of NRG-1β on glucose uptake and GLUT4 translocation in PA-treated C2C12 myotubes. In another study, NRG-1 did not affect the phosphorylation level of AKT in L6E9 myotubes (Canto et al., 2004). However, we found that NRG-1β significantly increased AKT phosphorylation in PA-treated C2C12 myotubes. It was reported that ceramides promoted IR *in vitro* by suppressing C2C12 and L6 myotube activities in different ways, probably reflecting differences in cell membrane structures or compositions (Mahfouz et al., 2014). However, further work is needed. In our study, we found that NRG-1β increased glucose uptake and GLUT4 translocation in PA-treated C2C12 myotubes *via* the PI3K/AKT signaling pathway.

We then studied the effect of NRG-1β on T2DM mice. In our study, NRG-1β improved diabetes-induced weight loss and diabetes-induced hyperglycemia obviously. Glucose is an important fuel for skeletal muscle, entering the cells *via* GLUT4, which is transferred from intracellular storage depots to the plasma membrane upon muscle contraction (Richter and Hargreaves, 2013). Previous studied reported that NRG-1 increased insulin sensitivity in normal skeletal muscle cells and improved glucose tolerance in db/db mice by liver regulatory action (Cantó et al., 2007; Ennequin et al., 2015; Caillaud et al., 2016; López-Soldado et al., 2016; Ennequin et al., 2020; Gumà et al., 2020). However, they only evaluated systematic IR; neither glucose uptake nor GLUT4 expression/translocation in the gastrocnemius was measured. In our study, we found that NRG-1β promoted the translocation of GLUT4 to the plasma membrane in gastrocnemius tissues of T2DM mice; this may improve the skeletal muscle IR. A further study should explore the mechanism by which NRG-1β affects skeletal muscle IR in T2DM mice.

In conclusion, NRG-1β increased glucose uptake and promoted GLUT4 translocation to the plasma membrane in PA-treated C2C12 myotubes by activating the PI3K/AKT signaling pathway. NRG-1β improved diabetes-induced weight loss and diabetes-induced hyperglycemia of T2DM mice compared to controls. Moreover, NRG-1β increased GLUT4 translocation in the gastrocnemius of T2DM mice.

## Data Availability

The original contributions presented in the study are included in the article/Supplementary Material, further inquiries can be directed to the corresponding authors.
