# Supplementary material for: Neuregulin-1β increases glucose uptake and promotes GLUT4 translocation in palmitate-treated C2C12 myotubes by activating PI3K/AKT signaling pathway
Source: Front Pharmacol. 2023 Jan 10;13:1066279. doi: 10.3389/fphar.2022.1066279 (PMC9871240; doi:10.3389/fphar.2022.1066279)
Supplement: Supplementary file 1 [file Image1.pdf]

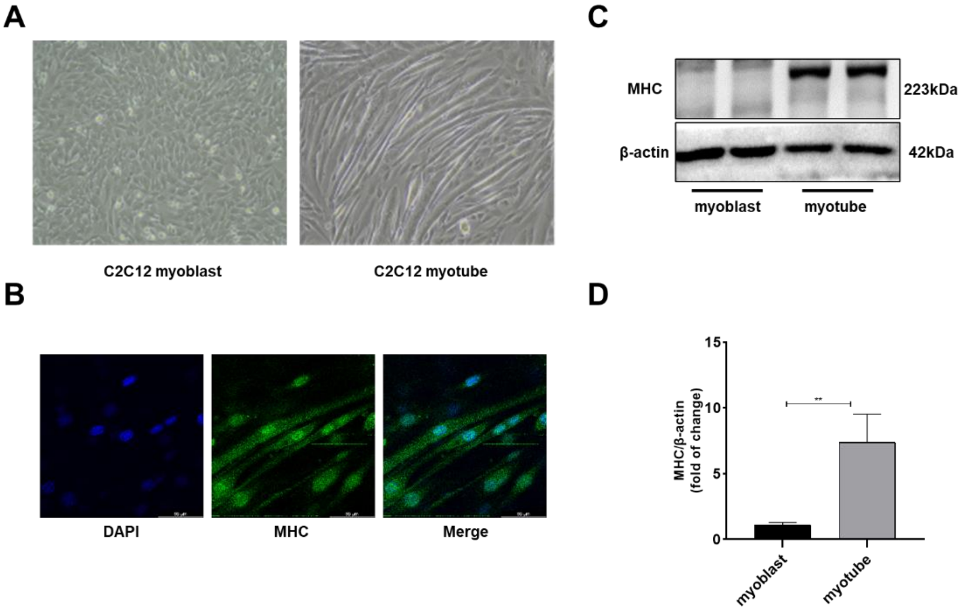

Supplementary Figure 1. Differentiation of C2C12 myotubes. Light microscopy (A) and immunofluorescence (B) showed myotube-like multi-nuclear structure. Scale bar: 30 μm. The protein marker MHC was detected via Western blotting (C-D). The data are presented as the mean ± SEM. $^{**}P < 0.01$.
